# Analysis of factors affecting the accuracy of colposcopic diagnosis of cervical lesions: a retrospective cohort study

**DOI:** 10.3389/fmed.2024.1462079

**Published:** 2024-10-16

**Authors:** Yuqing Chu, Qi Chen, Ruixue Liu, Xu Zhou, Meijing Bao, Hong Wang, Yang Lin

**Affiliations:** Department of Gynecology and Obstetrics, The Second Hospital of Jilin University, Changchun, China

**Keywords:** colposcopy, cervical cancer screening, HPV, cervical precancer, diagnostic accuracy

## Abstract

**Background:**

Cervical cancer is a leading cause of cancer-related deaths among women. However, in developing countries, the primary focus for cervical cancer prevention and control remains on the timely detection and treatment of precancerous lesions. This study aims to evaluate the accuracy of colposcopic diagnosis of cervical intraepithelial lesions and analyze the factors influencing its accuracy.

**Methods:**

This study collected data from 512 eligible patients who visited the gynecology outpatient clinic of the Second Hospital of Jilin University from January 2022 to August 2023. The final diagnosis was based on the highest grade observed in both biopsy pathology and postoperative pathology. A self-controlled, retrospective analysis was conducted to evaluate the accuracy of colposcopic diagnosis. Univariate analysis was used to assess the impact of factors such as menopausal status, duration of menopause, high-risk human papillomavirus (HR-HPV) genotypes, and transformation zone (TZ) types on the accuracy of colposcopic diagnosis of cervical lesions.

**Result:**

The overall colposcopy diagnostic concordance rate was 78.71%. The concordance rates between the premenopausal and postmenopausal groups differed significantly (*χ*^2^ = 75.450, *p* < 0.05). The HPV16/18 positive group and the non 16/18 HR-HPV positive group also showed a significant difference in concordance rates (*χ*^2^ = 5.890, *p* < 0.05). There were significant differences in concordance rates between TZ2 and TZ3 (*χ*^2^ = 10.585, *p* < 0.05), as well as between TZ1 and TZ3 (*χ*^2^ = 14.607, *p* < 0.05).

**Conclusion:**

Factors such as menopausal status, duration of menopause, HR-HPV genotypes, and TZ types influence the accuracy of colposcopic diagnosis. Therefore, a comprehensive evaluation incorporating these factors should be performed in clinical practice to enhance diagnostic accuracy.

## 1 Introduction

Cervical cancer is one of the most prevalent malignant tumors in the female reproductive system and the fourth leading cause of cancer-related deaths among women (1). It poses a significant threat to women’s health, exacerbating psychological and economic burdens on patients. Therefore, the prevention and treatment of cervical cancer are crucial.

Extensive research has established that persistent infection with high-risk human papillomavirus (HR-HPV) is the principal cause of precancerous lesions and cervical cancer. Preventive measures, including the HPV vaccine, have been developed (2). In developed countries, the widespread use of the HPV vaccine and cervical cancer screening has effectively reduced the incidence and mortality of cervical cancer (3). However, in developing countries where the HPV vaccine is not yet widely available, cervical cancer prevention and control efforts remain focused on the timely detection and appropriate treatment of precancerous lesions to prevent their progression to cervical cancer (4).

Studies indicate that the development of cervical cancer is a gradual process, typically requiring at least 8–12 years for precancerous lesions to progress to cervical cancer. Early clinical symptoms of cervical intraepithelial neoplasia and early cervical cancer are often very subtle (5). Therefore, cervical cancer screening is of paramount importance. The ultimate goal of prevention and treatment is not merely the timely detection of precancerous lesions and cervical cancer, but also the appropriate treatment and management following timely diagnosis. This approach is crucial for fundamentally reducing the incidence and mortality rates of cervical cancer.

The implementation of the “three-step” screening method for cervical cancer is now well-established (6). Colposcopy, which magnifies the observation area and utilizes acetic acid and Lugol’s iodine solution, can preliminarily locate and assess lesions, guiding biopsy sites. Colposcopy-guided cervical biopsy is regarded as the “gold standard” for diagnosing cervical abnormalities (7). Thus, colposcopy is indispensable.

Research indicates that the accuracy of colposcopic diagnosis and colposcopy-guided cervical biopsy for diagnosing cervical intraepithelial neoplasia and cervical cancer varies among different hospitals (8). Factors such as the type of transformation zone (TZ), menopausal status, and HR-HPV infection can affect the accuracy of colposcopic diagnosis and colposcopy-guided cervical biopsy (9). Therefore, identifying factors that impact the accuracy of colposcopy is crucial for improving diagnostic accuracy, providing clinicians with more reliable references for diagnosing and treating cervical abnormalities. Enhancing diagnostic and treatment accuracy will help avoid missed diagnoses of cervical lesions and unnecessary examinations and treatments for low-risk individuals.

## 2 Materials and methods

### 2.1 Study population

**Figure 1:**
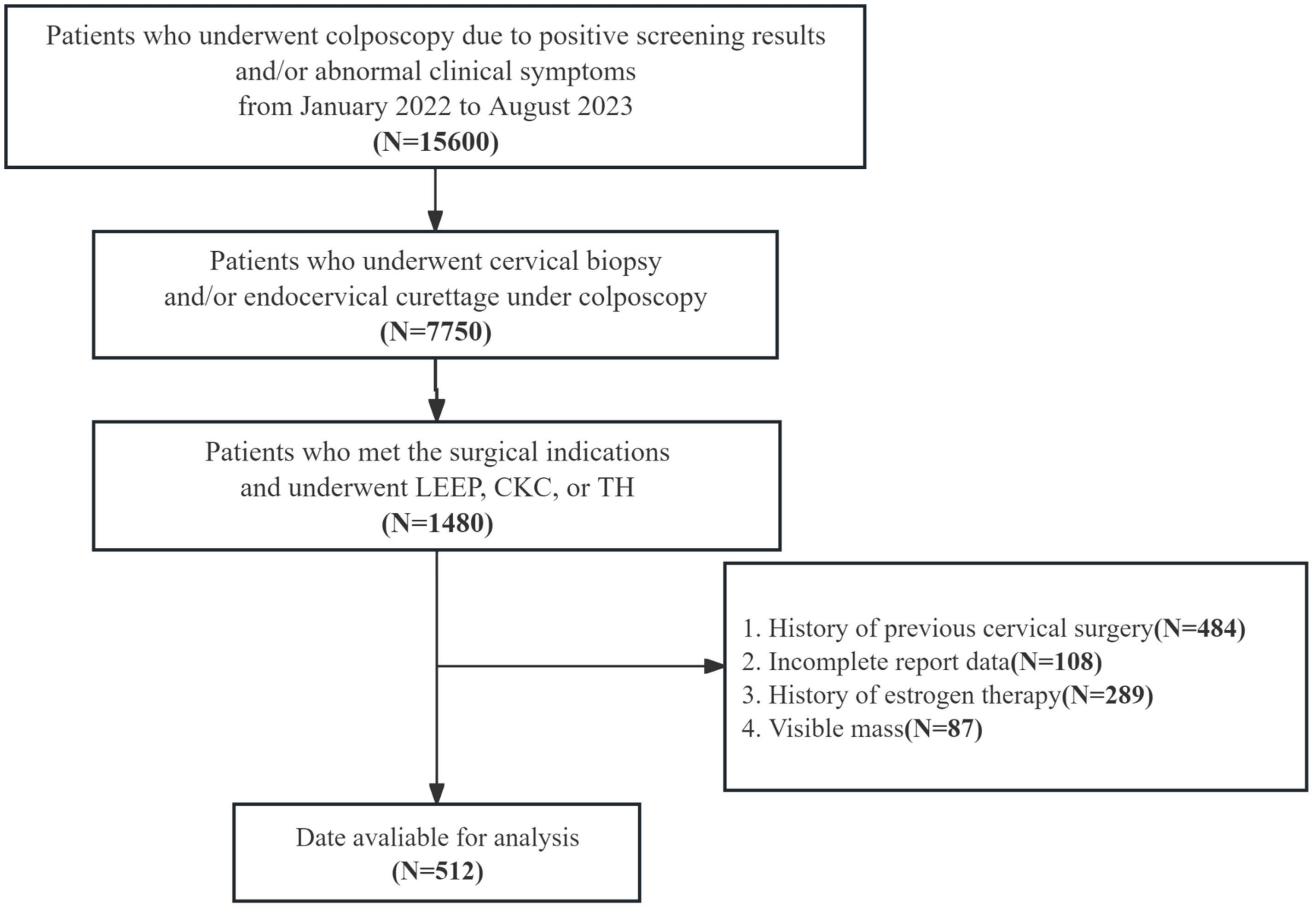
Flowchart illustrating the selection of study population. LEEP, loop electrosurgical excision procedure; CKC, cold knife conization; TH, total hysterectomy.

This study is a retrospective analysis of 512 patients who visited the gynecology outpatient clinic at the Second Hospital of Jilin University from January 2022 to August 2023. The inclusion criteria were: patients met the indications for colposcopy, had complete HPV and Thinprep cytologic test (TCT) results, and were diagnosed via colposcopy with normal, cervical intraepithelial neoplasia grade 1 (CIN1), or cervical intraepithelial neoplasia grade 2 or higher (CIN2+). All patients underwent colposcopic cervical biopsy and/or endocervical curettage. Based on pathological results indicating the need for surgical intervention, they underwent a loop electrosurgical excision procedure (LEEP), cold knife conization (CKC), or total hysterectomy. Patients who had used oral or local estrogen, or had a history of cervical physical treatment or surgery, were excluded. The flowchart selection of study population is depicted in Figure 1. This study adheres to the Declaration of Helsinki and was approved by the Institutional Review Board of the Second Hospital of Jilin University [(2024) Annual Review No. (082)]. Because this retrospective analysis is based on anonymized data, individual informed consent was not required.

### 2.2 Liquid-based cytology and HPV testing

After fully exposing the cervix, excess secretions are carefully removed using sterile cotton balls. A specialized sampling brush is subsequently employed to collect cervical cell specimens, which are subsequently placed in a specimen vial and sent for analysis. The results of the liquid-based cytology test are evaluated according to the 2014 Bethesda System (TBS) diagnostic criteria endorsed by the International Cancer Association (10). High-risk HPV testing is conducted utilizing the second-generation hybrid capture method (Hybrid Capture II, HC2), including both viral load measurement and viral typing. The viral load is quantified as the ratio of relative light units (RLU) to the standard positive control (CO). Viral typing includes HPV16/18 positivity as well as the other 12 high-risk types (including HPV31/33/35/39/45/51/52/56/58/59/66/68) (11).

### 2.3 Colposcopy and histological diagnosis

Colposcopy is a visual examination technique employed to assess the cervix, vaginal wall, vulva, and perianal epithelium. It involves the use of appropriate illumination and magnification, combined with acetic acid and compound iodine tests, to identify abnormal lesions. Biopsies are performed on suspicious areas under colposcopic guidance for further diagnostic evaluation. Colposcopic findings are classified based on the terminology established by the International Federation for Cervical Pathology and Colposcopy (IFCPC) in 2011 (12). To minimize variability due to differing experience levels, all examinations were performed by senior colposcopists, each with over 10 years of experience and comparable diagnostic expertise. In this study, both colposcopic diagnoses and pathological results were classified into three categories: Normal, CIN1, and CIN2+ (which includes CIN2, CIN3, and squamous cell carcinoma). The final diagnostic criterion was determined by the higher-grade finding between the colposcopic biopsy and postoperative pathology. A diagnosis was considered concordant when the colposcopic findings matched the highest pathological result. If the colposcopic diagnosis was lower than the highest pathology grade, it was considered under-diagnosed; if higher, it was classified as over-diagnosed.

### 2.4 Statistical analysis

Data are presented as frequencies and percentages (%). Statistical analysis is conducted using SPSS 26.0 software, with the Chi-square test applied to assess the impact of relevant factors on the accuracy of colposcopic diagnosis. A *p*-value <0.05 indicates statistical significance.

## 3 Results

### 3.1 Clinical characteristics of study population

The clinical information of the colposcopy population is presented in Table 1. This study included 512 subjects, with ages ranging from 19 to 74 years, with a median age of 37 years. The age distribution was as follows: 34 cases (6.64%) were under 25 years old, 66 cases (12.89%) were between 25 and 29 years old, 191 cases (37.31%) were between 30 and 39 years old, 111 cases (21.68%) were between 40 and 49 years old, and 110 cases (21.48%) were 50 years or older. Regarding menopausal status, 346 cases (67.58%) were premenopausal, and 166 cases (32.42%) were postmenopausal. HPV testing revealed that 340 cases (66.41%) were positive for HPV16/18, while 172 cases (33.59%) were positive for non-16/18 HR-HPV. The transformation zone (TZ) was classified as TZ1 in 132 cases (25.78%), TZ2 in 126 cases (24.62%), and TZ3 in 254 cases (49.61%). Colposcopic diagnosis identified 12 cases (2.34%) with a normal cervix, 173 cases (33.79%) with CIN1, and 327 cases (63.87%) with CIN2+. The highest pathological diagnosis results included 17 cases (3.32%) with a normal cervix, 136 cases (26.56%) with CIN1, 359 cases (70.12%) with CIN2+.

**Table 1:** Clinical characteristics of study population.

Variables	*n* (%)
Total	*N* = 512
Age	
<25	34 (6.64)
25–29	66 (12.89)
30–39	191 (37.31)
40–49	111 (21.68)
≥50	110 (21.48)
Menopausal status	
Premenopausal	346 (67.58)
Postmenopausal	166 (32.42)
HPV status	
HPV16/18	340 (66.41)
Non-16/18 HR-HPV	172 (33.59)
Transformation zone	
TZ1	132 (25.78)
TZ2	126 (24.62)
TZ3	254 (49.61)
Colposcopic diagnostic	
Normal, *n* (%)	12 (2.34)
CIN1, *n* (%)	173 (33.79)
CIN2+, *n* (%)	327 (63.87)
Highest pathological diagnosis	
Normal, *n* (%)	17 (3.32)
CIN1, *n* (%)	136 (26.56)
CIN2+, *n* (%)	359 (70.12)

CIN1, cervical intraepithelial neoplasia grade 1; CIN2+, cervical intraepithelial neoplasia grade 2 or worse.

### 3.2 Consistency between colposcopic diagnosis and histopathology

Table 2 summarizes the colposcopic diagnosis of cervical lesions: 12 cases (2.34%) were classified as negative, 173 cases (33.79%) as CIN1, and 327 cases (63.87%) as CIN2+. The overall concordance rate of colposcopic diagnosis was 78.71% (403/512), with a non-concordance rate of 21.29% (109/512). Among the non-concordant cases, the rate of underdiagnosis was 73.39% (80/109) and the rate of overdiagnosis was 26.61% (29/109). We found that the concordance rate for colposcopically diagnosed CIN1 was 65.32% (113/173), while for CIN2+ it was 86.54% (283/327). The difference between these rates was statistically significant (*χ*^2^ = 30.945, *p* < 0.05), indicating that the concordance rate for CIN2+ was significantly higher than for CIN1.

**Table 2:** Comparison of colposcopic diagnosis with highest pathological diagnosis.

Colposcopic diagnosis	Highest pathological diagnosis			Total	Concordance rate (%)	*χ* ^2^	*p*-value
	Underdiagnosis	Concordance	Overdiagnosis				
Normal	5	7	0	12	58.33		
CIN1	50	113	10	173	65.32	30.945	<0.05
CIN2+	25	283	19	327	86.54		
Total	80	403	29	512	78.71		

CIN1, cervical intraepithelial neoplasia grade 1; CIN2+, cervical intraepithelial neoplasia grade 2 or worse.

### 3.3 Analysis of factors affecting the accuracy of colposcopic diagnosis

#### 3.3.1 The influence of menopausal status on the accuracy of colposcopic diagnosis

Based on the patients’ menopausal status, the 512 cases were categorized into premenopausal and postmenopausal groups. The premenopausal group consisted of 346 cases (67.58%). Among these, the colposcopic diagnosis results were concordant with the highest pathological diagnosis in 310 cases and discordant in 36 cases, yielding a concordance rate of 89.60% (310/346). The postmenopausal group comprised 166 cases (32.42%), with colposcopic diagnosis results concordant with the highest pathological diagnosis in 93 cases and discordant in 73 cases, resulting in a concordance rate of 56.02% (93/166). As shown in Table 3, the comparison of concordance rates between the premenopausal and postmenopausal groups revealed a statistically significant difference (*χ*^2^ = 75.450, *p* < 0.05). The concordance rate of colposcopic diagnosis in the premenopausal group proved to be significantly higher than that in the postmenopausal group.

**Table 3:** Impact of menopausal status on colposcopic diagnostic accuracy.

Menopausal status	Concordance	Non-concordance	Total	Concordance rate (%)	*χ* ^2^	*p*-value
Premenopausal	310	36	346	89.60	75.45	<0.05
Postmenopausal	93	73	166	56.02		
Total	403	109	512			

#### 3.3.2 The influence of menopause duration on the accuracy of colposcopic diagnosis

The 166 postmenopausal patients were divided into two groups based on the duration of menopause: ≤2 years and >2 years. There were 54 cases (32.53%) in the menopause ≤2 years group. Colposcopy impressions were compared with the highest pathological diagnosis, revealing 37 consistent and 17 inconsistent cases, resulting in an accuracy rate of 68.52% (37/54). In the menopause >2 years group, there were 112 cases (67.47%). In this group, 56 cases were consistent and 56 were inconsistent when comparing colposcopy impressions with the highest pathological diagnosis, resulting in an accuracy rate of 50.00% (56/112). As shown in Table 4, the comparison of colposcopic accuracy between the two groups revealed a statistically significant difference (*χ*^2^ = 5.071, *p* < 0.05). The colposcopy accuracy rate in the menopause ≤2 years group was significantly higher than that in the menopause >2 years group.

**Table 4:** Impact of menopause duration on the colposcopy diagnosis accuracy.

Menopause duration	Concordance	Non-concordance	Total	Concordance rate (%)	*χ* ^2^	*p*-value
≤2 years	37	17	54	68.52	5.071	<0.05
>2 years	56	56	112	50.00		
Total	93	73	166	56.02		

#### 3.3.3 The influence of high-risk HPV genotypes on the accuracy of colposcopic diagnosis

Based on HR-HPV genotypes, 512 patients were divided into two groups: the HPV16/18 positive group and the non-HPV16/18 HR-HPV positive group. The HPV16/18 positive group comprised 172 cases (33.59%), with 146 cases where the colposcopic diagnosis matched the highest pathological diagnosis and 26 cases where it did not, resulting in a concordance rate of 84.88% (146/172). The non-HPV16/18 HR-HPV positive group consisted of 340 cases (66.41%), with 257 cases where the colposcopic diagnosis matched the highest pathological diagnosis and 83 cases where it did not, yielding a concordance rate of 75.59% (257/340). As shown in Table 5, the comparison of the two groups revealed a statistically significant difference (*χ*^2^ = 5.890, *p* < 0.05), indicating that the concordance rate of colposcopic diagnosis in the HPV16/18 positive group was significantly higher than that in the non-HPV16/18 positive group.

**Table 5:** Impact of high-risk HPV subtypes on colposcopic diagnostic accuracy.

HPV status	Concordance	Non-concordance	Total	Concordance rate (%)	*χ* ^2^	*p*-value
HPV16/18	146	26	172	84.88	5.890	<0.05
Non-16/18 HR-HPV	257	83	340	75.59		
Total	403	109	512			

#### 3.3.4 The influence of transformation zone type on the accuracy of colposcopic diagnosis

Based on the types of transformation zones, 512 patients were divided into three groups: Type I, Type II, and Type III transformation zones. There were 132 cases in the Type I group (25.78%), 126 cases in the Type II group (24.61%), and 254 cases in the Type III group (49.61%). As shown in Table 6, when comparing colposcopic diagnosis results with the highest pathological diagnosis results, the Type I group had 116 cases that were consistent, resulting in a concordance rate of 87.88% (116/132). The Type II group had 108 cases that were consistent, with a concordance rate of 85.71% (108/126). The Type III group had 179 cases that were consistent, yielding a concordance rate of 70.47% (179/254).

**Table 6:** Impact of transformation zone on colposcopic diagnostic accuracy.

TZ	Concordance	Non-concordance	Total	Concordance rate (%)
TZ1	116	16	132	87.88
TZ2	108	18	126	85.71
TZ3	179	75	254	70.47
Total	403	109	512	

TZ, transformation zone.

When comparing the concordance rates of colposcopic diagnosis between the groups, no significant difference was observed between the Type I and Type II groups (*χ*^2^ = 0.264, *p* > 0.05), indicating that the concordance rates between these two groups were not significantly different. However, the comparison between the Type II and Type III groups revealed a statistically significant difference (*χ*^2^ = 10.585, *p* < 0.05), with the Type II group having a significantly higher concordance rate than the Type III group. Similarly, the comparison between the Type I and Type III groups showed a statistically significant difference (*χ*^2^ = 14.607, *p* < 0.05), with the Type I group having a significantly higher concordance rate than the Type III group. In summary, as shown in Table 7, the concordance rates of colposcopic diagnosis for Type I and Type II transformation zones are significantly higher than those for Type III transformation zones.

**Table 7:** Pairwise comparison.

Group comparison	*χ* ^2^	*p*-value	Statistical significance
TZ1 and TZ2	0.264	0.607	Not Significant
TZ2 and TZ3	10.585	<0.05	Significant
TZ1 and TZ3	14.607	<0.05	Significant

TZ, transformation zone.

## 4 Discussion

Cervical cancer is the fourth leading cause of cancer-related deaths among women; therefore, its prevention and treatment are imperative. In clinical practice, colposcopy overdiagnosis can lead to overtreatment, imposing significant mental, physical, and economic burdens on patients. Conversely, underdiagnosis can result in missed diagnoses, thereby causing patients to miss the optimal window for early detection and treatment (13). Therefore, analyzing factors affecting colposcopy accuracy is crucial for enhancing diagnostic precision, thus providing valuable insights for clinicians in diagnosing and treating cervical lesions. This approach helps avoid missed diagnoses and reduces unnecessary examinations and treatments in low-risk populations.

### 4.1 Analysis of colposcopy diagnostic accuracy

In this study, we conducted a retrospective analysis of clinical data from 512 patients to evaluate the accuracy of colposcopic diagnosis and colposcopically-guided cervical biopsy. We also analyzed factors affecting the accuracy of colposcopic diagnosis of cervical intraepithelial lesions, aiming to reduce the impact of these factors on diagnostic accuracy, decrease the rates of missed and misdiagnosis, and enhance overall diagnostic precision. When comparing the colposcopy impressions of cervical intraepithelial lesions with the final pathological results, the overall concordance rate was 78.71% (403/512), and the overall discordance rate was 21.29% (109/512). Among these cases, 80 were underdiagnosis and 29 were overdiagnosis, similar to studies conducted in other cities in China (14, 15). In our study, the sensitivity of colposcopic diagnosis was approximately 93.18% (396/425), and the specificity was about 58.33% (7/12). Reviewing previous literature, the sensitivity and specificity of colposcopic examination for cervical lesions have varied. A systematic review and meta-analysis evaluated the colposcopic diagnosis using the International Federation of Cervical Pathology and Colposcopy (IFCPC) 2011 terminology. The results showed that for LSIL and above, the overall sensitivity was 92% (95% CI 0.88–0.95) and specificity was 51% (95% CI 0.43–0.59). For HSIL and above, the overall sensitivity was 68% (95% CI 0.58–0.76) and specificity was 93% (95% CI 0.88–0.96) (16). Additionally, an Indian study reported a colposcopic sensitivity of 96.4% and specificity of 39.53% (17). These findings indicate that while colposcopy is highly sensitive in detecting cervical lesions, its specificity can be relatively low, potentially leading to a higher rate of false positives. These results highlight the importance of continuously improving colposcopic diagnostic techniques and training to enhance diagnostic accuracy.

**Figure 2:**
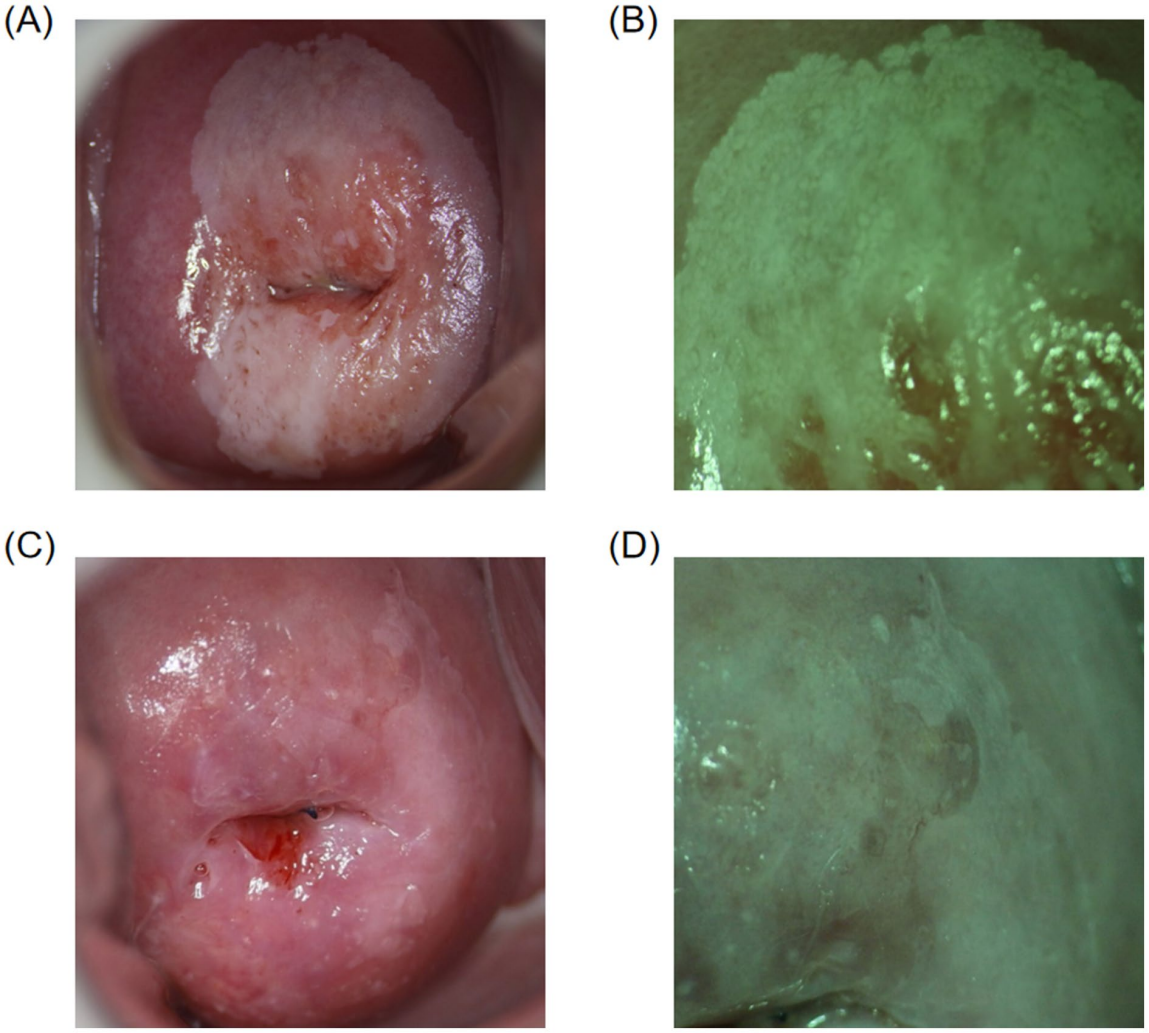
Colposcopic images. **(A,B)** Colposcopically diagnosed as CIN2+, with final pathology confirming the diagnosis. Thick acetowhite epithelium with clear boundaries is observed on the cervical surface after acetic acid application, involving multiple quadrants, raised with mosaic patterns and glandular openings. **(C,D)** Colposcopically diagnosed as CIN1, with final pathology confirming the diagnosis. Thin acetowhite epithelium with less clear boundaries is observed on the cervical surface after acetic acid application.

In this study, the concordance rate for colposcopy impressions of CIN1 was 65.32% (113/173), while for CIN2+ it was 86.54% (283/327). A Chi-square test indicated that this difference was statistically significant (*p* < 0.05). This indicates that the concordance rate for colposcopy impressions of CIN2+ is significantly higher than that of CIN1, which is consistent with the conclusions of related studies. For example, a systematic review and meta-analysis found that the sensitivity and specificity for detecting HSIL and above are higher than for LSIL (16). Additionally, a study of 4,778 colposcopy cases showed that the concordance rate for HSIL was 59.5%, significantly higher than the 43.4% concordance rate for LSIL (18). High-grade cervical intraepithelial lesions exhibit more distinct colposcopic image characteristics, such as large areas of thick acetowhite epithelium, extensive involvement, rapid appearance and slow disappearance, clear boundaries, and associated features like coarse punctation, coarse mosaic patterns, and glandular openings, making them easier for clinicians to identify (Figures 2A,B). Conversely, low-grade cervical intraepithelial lesions are more dispersed and have less distinct features compared to normal tissue under colposcopy, making diagnosis more challenging and prone to missed diagnoses (Figures 2C,D). Therefore, it is crucial for clinicians to carefully and comprehensively observe the images, integrate image analysis capabilities, while considering the patient’s medical history (19). By continuously accumulating diagnostic experience, improving technical skills, and staying updated with the latest research findings and guideline updates, clinicians can more effectively enhance diagnostic accuracy and reduce the rate of missed diagnoses, thereby providing more accurate and effective treatment recommendations for patients.

### 4.2 Analysis of factors influencing colposcopy diagnostic accuracy

#### 4.2.1 Menopausal status and duration

This study divided 512 patients into premenopausal and postmenopausal groups based on their menopausal status. The colposcopy diagnostic accuracy rate for the premenopausal group was 89.60% (310/346), whereas it was 56.02% (93/166) for the postmenopausal group. A comparison of the colposcopy diagnostic accuracy rates between the two groups revealed a statistically significant difference (*χ*^2^ = 75.450, *p* < 0.05), demonstrating that the colposcopy diagnostic accuracy rate was significantly higher in the premenopausal group than in the postmenopausal group. This result is consistent with other related studies. Bo and Ge (20) found that the accuracy of colposcopy diagnosis and the satisfaction with colposcopic examinations were higher in the premenopausal group compared to the postmenopausal group. Chen’s et al. (21) research indicated that women over the age of 45 are at a higher risk of missed diagnosis during colposcopic screening (*p* = 0.0195), with a corresponding decrease in diagnostic accuracy rates as age increases.

This study further analyzed the impact of menopausal duration on the accuracy of colposcopy diagnosis. The results showed that the colposcopy diagnostic accuracy rate was 68.52% (37/54) for the group with menopause duration of ≤2 years, compared to 50.00% (56/112) for the group with menopause duration of >2 years. The comparison between the two groups revealed a statistically significant difference (*χ*^2^ = 5.071, *p* < 0.05), demonstrating that the colposcopy diagnostic accuracy rate was significantly higher in the group with menopause duration of ≤2 years compared to the group with menopause duration of >2 years. This result is consistent with related studies. Müller et al. (22) found that the agreement rate between colposcopically directed cervical biopsy pathology results and postoperative pathology results was correlated with patient age. The highest agreement rate, 90.7%, was observed in patients under 30 years old, while the lowest rate, 72.1%, was found in patients over 50 years old. Costa’s et al. (23) research showed that the rate of missed diagnoses in colposcopy increases with patient age and the prevalence of the invisible squamocolumnar junction, especially among patients over 50 years old, who are more prone to missed diagnoses.

**Figure 3:**
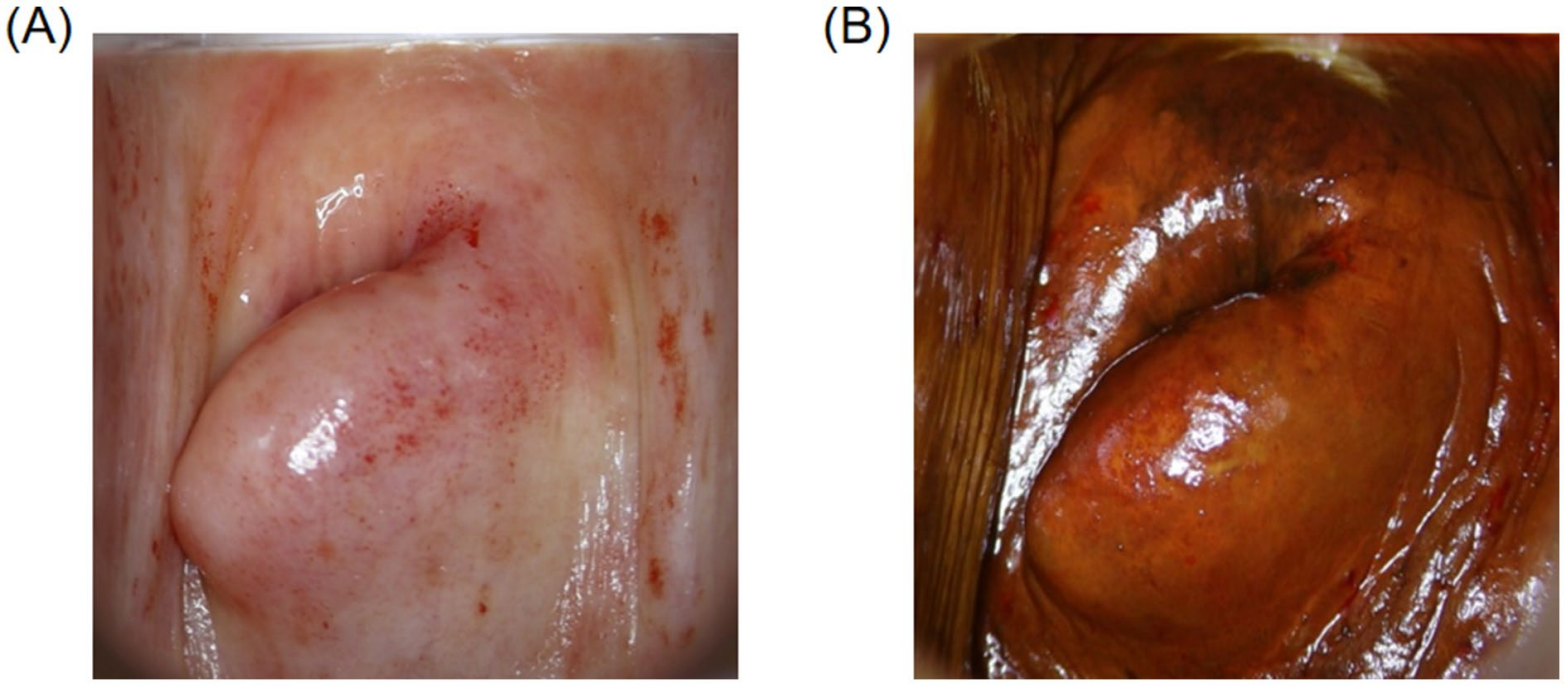
Colposcopic images. **(A)** Image of the cervix in a postmenopausal woman after acetic acid application. **(B)** Image of the cervix in a postmenopausal woman after Lugol’s iodine staining.

As women age and hormone levels decline, postmenopausal physiological changes present numerous challenges to the accuracy of colposcopic examinations. First, the cervix in postmenopausal women typically atrophies, and the squamocolumnar junction (SCJ) gradually migrates into the endocervical canal. Since the SCJ is a high-risk area for cervical lesions, when these lesions occur within the endocervical canal, they are difficult to visualize during colposcopy, thus increasing diagnostic difficulty. Additionally, with advancing age, vaginal atrophy and dryness may result in incomplete exposure of the examination field, which not only affects the visibility during the examination but also increases procedural difficulty and patient discomfort (24). In postmenopausal women, the squamous epithelium of the cervix and vagina becomes atrophic and thinner, leading to reduced acetic acid reactivity in lesion areas (Figure 3A). Furthermore, the glycogen content in the squamous epithelium decreases, causing Lugol’s iodine staining to cover a larger area, making lesions appear atypical and difficult to distinguish from normal tissue (Figure 3B), thereby increasing the risk of missed diagnoses and false negatives (25).

Despite the increased challenges associated with colposcopic examinations due to postmenopausal physiological changes, the significance of colposcopy in postmenopausal women remains critical. It has been demonstrated in Zappacosta’s et al. (26) research that cytological screening may not be entirely reliable in postmenopausal women, particularly in patients with persistent HPV infection. In this case report, a 79-year-old woman who had regularly undergone cervical cytological screening had normal cytological findings but was eventually diagnosed with HPV-53-associated cervical cancer. Thus, colposcopy remains essential in postmenopausal women.

In order to mitigate the negative impact of postmenopausal physiological changes on the accuracy of colposcopic examinations, appropriate clinical interventions should be implemented. First, a thorough assessment of the patient’s menopausal status, duration of menopause, and relevant medical history should be conducted prior to the examination. To improve diagnostic accuracy in postmenopausal women, short-term local estrogen therapy may be considered if no contraindications are present. The application of local estrogen can significantly increase the thickness and glycogen content of the cervical and vaginal squamous epithelium, thereby enhancing the differentiation between normal and abnormal tissues during acetic acid and Lugol’s iodine staining, which ultimately improves diagnostic accuracy (27). Studies have demonstrated that local estrogen intervention prior to colposcopic examination in postmenopausal women can significantly reduce atrophic changes, enhance the visibility of the examination field, and make the procedure more comprehensive (28). Thus, the application of local estrogen therapy prior to colposcopy in postmenopausal women serves as an effective strategy to enhance diagnostic accuracy and improve overall examination outcomes.

#### 4.2.2 High-risk HPV genotypes

In this study, based on HR-HPV genotypes, 512 patients were divided into two groups: the HPV16/18 positive group and the non-HPV16/18 HR-HPV positive group. The colposcopy diagnostic accuracy rates of the two groups were compared. The results showed that the colposcopy diagnostic accuracy rate was significantly higher in the HPV16/18 positive group compared to the non-HPV16/18 HR-HPV positive group (*χ*^2^ = 5.890, *p* < 0.05), demonstrating a statistically significant difference. Currently, many studies have reported conclusions consistent with this study. Stoler’s et al. (29) research, which included 737 patients, indicated that HPV16/18 positivity increases the accuracy of colposcopy. Nam’s et al. (30) study showed that the range and extent of lesions observed during colposcopy vary with the type of HR-HPV infection. Specifically, HPV16 is associated with more severe and extensive lesions, which aids in improving colposcopy accuracy. A large prospective clinical study based on initial HPV screening in the United States found that HPV16/18 positivity is more likely to be associated with CIN2+ lesions compared to other HR-HPV types (31). Additionally, other studies have indicated that HPV16 viral load can serve as a relevant biomarker for identifying women at high risk for cervical precancerous lesions (32).

The colposcopy diagnostic accuracy of the HPV16/18 positive group is significantly higher than that of the non-HPV16/18 HR-HPV positive group. The primary reason for this may be that HPV16/18 viruses produce E6 and E7 proteins, which have stronger pathogenic capabilities, playing a crucial role in the development of cervical lesions (33). E6 protein can bind to the P53 protein, leading to its degradation and thus inhibiting the cell’s ability to repair damaged DNA. E7 protein, by binding to Rb protein, causes Rb protein inactivation, resulting in the loss of cell proliferation regulation. The synergistic action of these two proteins makes cells prone to chromosomal mutations and uncontrolled proliferation, eventually leading to cancer (34). During colposcopy, the cellular changes caused by E6 and E7 proteins often exhibit distinct morphological characteristics, making the lesion areas easier to identify, thereby improving the diagnostic accuracy of colposcopy. However, the accuracy of colposcopy diagnosis not only depends on the visibility of the lesions but also on the subjective judgment of the physician. The widely recognized association between high-risk HPV infection and cervical precancerous lesions may influence colposcopy diagnostic decisions to some extent. Therefore, a rigorous and comprehensive analysis should be conducted in clinical practice to ensure the accuracy of colposcopy diagnosis.

Given the significant role of HPV E6/E7 proteins in cervical lesion progression, HPV E6/E7 mRNA expression can be assessed to measure the transcriptional activity of HPV oncogenes and their impact on host cells, thereby providing a basis for evaluating cervical lesions. HPV E6/E7 mRNA testing has been shown to offer higher specificity while maintaining comparable sensitivity to HPV DNA testing. For example, a study conducted by Zappacosta et al. (35) demonstrated that, in colposcopic follow-up, HPV E6/E7 mRNA testing exhibited higher specificity and positive predictive value compared to cytology combined with HPV DNA testing. This indicates that mRNA testing not only enhances the diagnostic accuracy of colposcopy but may also serve as a valuable tool for predicting residual or recurrent cervical disease, particularly in patients post-LEEP conization. These findings indicate that mRNA testing could become a valuable adjunct in future clinical practice, potentially improving the diagnostic effectiveness of colposcopy and reducing overtreatment risks.

#### 4.2.3 Transformation zone types

In this study, 512 patients were divided into TZ1, TZ2 and TZ3 based on the type of transformation zone, and comparisons were made between each pair. The results showed that the colposcopy diagnostic accuracy rates for both the TZ1 and TZ2 groups were significantly higher than those for the TZ3 group. These findings are consistent with related studies. Stuebs et al. (36) found that the accuracy and sensitivity for detecting HSIL were significantly higher for TZ1 and TZ2 compared to TZ3. Specifically, the accuracy rates for TZ1 and TZ2 were 75.4 and 70.9%, respectively, while the accuracy rate for TZ3 was only 62.2%. Li et al. (25) found that the type of transformation zone affects the accuracy of colposcopy diagnosis, with the TZ3 having the lowest diagnostic accuracy. Dan (37) showed that the positivity rate of cervical canal lesions increases as the visible range of the cervical transformation zone decreases, and the difficulty of colposcopy and cervical biopsy also increases, leading to missed diagnoses in colposcopy and cervical biopsy. Therefore, clinicians should pay attention to the type of transformation zone, particularly the location of the squamocolumnar junction (SCJ). If necessary, instruments can be used to help expose the area. For Type III transformation zones, a proactive approach, such as endocervical curettage, may be necessary to improve diagnostic accuracy.

## 5 Conclusion

In summary, we found that the colposcopy diagnostic accuracy in identifying CIN2+ is significantly higher compared to CIN1. Factors such as menopausal status, duration of menopause, high-risk HPV subtypes, and transformation zone type affect the accuracy of colposcopy diagnosis. Therefore, a comprehensive evaluation incorporating these factors should be performed in clinical practice to enhance diagnostic accuracy.

## Data Availability

The original contributions presented in the study are included in the article/supplementary material, further inquiries can be directed to the corresponding author.

## References

[1] Bray F Laversanne M Sung H Ferlay J Siegel RL Soerjomataram I. Global cancer statistics 2022: GLOBOCAN estimates of incidence and mortality worldwide for 36 cancers in 185 countries. CA Cancer J Clin. (2024) 74:229–63. doi: 10.3322/caac.21834, PMID: 38572751

[2] Wheeler CM. Advances in primary and secondary interventions for cervical cancer: human papillomavirus prophylactic vaccines and testing. Nat Clin Pract Oncol. (2007) 4:224–35. doi: 10.1038/ncponc0770 17392713

[3] Marc Arbyn P Elisabete Weiderpass P Laia Bruni M Silvia De Sanjosé P Mona Saraiya M Jacques Ferlay I. Estimates of incidence and mortality of cervical cancer in 2018: a worldwide analysis. Lancet Glob Health. (2020) 8:e191–203. doi: 10.1016/s2214-109x(19)30482-6 31812369 PMC7025157

[4] Ginsburg O Bray F Coleman MP Vanderpuye V Eniu A Kotha SR. The global burden of women’s cancers: a grand challenge in global health. Lancet. (2017) 389:847–60. doi: 10.1016/s0140-6736(16)31392-7 27814965 PMC6191029

[5] Castle PE Maza M. Prophylactic HPV vaccination: past, present, and future. Epidemiol Infect. (2016) 144:449–68. doi: 10.1017/S0950268815002198 26429676

[6] Ronco G Dillner J Elfström KM. Efficacy of HPV-based screening for prevention of invasive cervical cancer: follow-up of four European randomised controlled trials. Lancet. (2014) 383:524–32. doi: 10.1016/S0140-6736(13)62218-7 24192252

[7] Massad LS Einstein MH Huh WK Katki HA Kinney WK Schiffman M. 2012 updated consensus guidelines for the management of abnormal cervical cancer screening tests and cancer precursors. Obstet Gynecol. (2013) 121:829–46. doi: 10.1097/AOG.0b013e3182883a34 23635684

[8] Offen D Shtaif B Hadad D Weizman A Melamed E Gil-Ad I. Protective effect of insulin-like-growth-factor-1 against dopamine-induced neurotoxicity in human and rodent neuronal cultures: possible implications for Parkinson’s disease. Neurosci Lett. (2001) 316:129–32. doi: 10.1016/s0304-3940(01)02344-8 11744219

[9] Wentzensen N Massad LS Mayeaux EJ Khan MJ Waxman AG Einstein MH. Evidence-based consensus recommendations for colposcopy practice for cervical cancer prevention in the United States. J Low Genit Tract Dis. (2017) 21:216–22. doi: 10.1097/lgt.0000000000000322 28953109

[10] Nayar R Wilbur DC. The Bethesda system for reporting cervical cytology: a historical perspective. Acta Cytol. (2017) 61:359–72. doi: 10.1159/000477556, PMID: 28693017

[11] Cheah P-L Looi L-M Sivanesaratnam V. Human papillomavirus in cervical cancers of Malaysians. J Obstet Gynaecol Res. (2011) 37:489–95. doi: 10.1111/j.1447-0756.2010.01386.x 21349124

[12] Bornstein J Sideri M Tatti S Walker P Prendiville W Haefner HK. 2011 terminology of the vulva of the International Federation for Cervical Pathology and Colposcopy. J Low Genit Tract Dis. (2012) 16:290–5. doi: 10.1097/LGT.0b013e31825934c7 22659778

[13] Wegwarth O Gigerenzer G. Overdiagnosis and overtreatment: evaluation of what physicians tell their patients about screening harms. JAMA Intern Med. (2013) 173:2086–7. doi: 10.1001/jamainternmed.2013.10363, PMID: 24145597

[14] Li J Wang W Yang P Chen J Dai Q Hua P. Analysis of the agreement between colposcopic impression and histopathological diagnosis of cervical biopsy in a single tertiary center of Chengdu. Arch Gynecol Obstet. (2021) 304:1033–41. doi: 10.1007/s00404-021-06012-y 33683424

[15] Li Y Duan X Sui L Fengying X Shuifang X Zhang H. Closer to a uniform language in colposcopy: study on the potential application of 2011 International Federation for Cervical Pathology and Colposcopy Terminology in Clinical Practice. Biomed Res Int. (2017) 2017:8984516. doi: 10.1155/2017/8984516 28626767 PMC5463115

[16] Qin D Bai A Xue P Seery S Wang J Mendez MJG. Colposcopic accuracy in diagnosing squamous intraepithelial lesions: a systematic review and meta-analysis of the International Federation of Cervical Pathology and Colposcopy 2011 terminology. BMC Cancer. (2023) 23:187. doi: 10.1186/s12885-023-10648-1, PMID: 36823557 PMC9951444

[17] Mehta R Yadav R Singh CP. Comparison of conventional pap smear, colposcopy, and HPV testing in diagnosis of CIN. Indian J Gynecol Oncolog. (2021) 19:33. doi: 10.1007/s40944-021-00512-0

[18] Lei L Zhang L Zheng Y Ma W Liu F Li D. Clinical analysis of 314 patients with high-grade squamous intraepithelial lesion who underwent total hysterectomy directly: a multi-center, retrospective cohort study. BMC Cancer. (2024) 24:575. doi: 10.1186/s12885-024-12342-2, PMID: 38724921 PMC11080298

[19] Massad LS Einstein MH Huh WK Katki HA Kinney WK Schiffman M. 2012 updated consensus guidelines for the management of abnormal cervical cancer screening tests and cancer precursors. J Low Genit Tract Dis. (2013) 17:S1–S27. doi: 10.1097/LGT.0b013e318287d329 23519301

[20] Bo ZXM Ge M. Diagnosis and treatment of high-grade cervical intraepithelial neoplasia in postmenopausal women by LEEP. Int J Gynecol Obstet. (2020) 47:33–6.

[21] Chen RJ Chang DY Yen ML Lee EF Chow SN Huang SC. Independent clinical factors which correlate with failures in diagnosing early cervical cancer. Gynecol Oncol. (1995) 58:356–61. doi: 10.1006/gyno.1995.1242, PMID: 7672701

[22] Müller K Soergel P Hillemanns P Jentschke M. Accuracy of colposcopically guided diagnostic methods for the detection of cervical intraepithelial neoplasia. Geburtshilfe Frauenheilkd. (2016) 76:182–7. doi: 10.1055/s-0041-111504, PMID: 26941452 PMC4771495

[23] Costa S De Nuzzo M Infante FE Bonavita B Marinelli M Rubino A. Disease persistence in patients with cervical intraepithelial neoplasia undergoing electrosurgical conization. Gynecol Oncol. (2002) 85:119–24. doi: 10.1006/gyno.2001.6579 11925130

[24] Benini V Ruffolo AF Casiraghi A Degliuomini RS Frigerio M Braga A. New innovations for the treatment of vulvovaginal atrophy: an up-to-date review. Medicina. (2022) 58:770. doi: 10.3390/medicina58060770, PMID: 35744033 PMC9230595

[25] Li XX Zhao YZ Xiang FF Zhang XP Chen ZX Zhang MZ. Evaluation of the diagnostic performance of colposcopy in the detection of cervical high-grade squamous intraepithelial lesions among women with transformation zone type 3. BMC Cancer. (2024) 24:–381. doi: 10.1186/s12885-024-12156-2, PMID: 38528547 PMC10964607

[26] Zappacosta R Lattanzio G Viola P Ianieri MM Gatta DM Rosini S. A very rare case of HPV-53-related cervical cancer, in a 79-year-old woman with a previous history of negative Pap cytology. Clin Interv Aging. (2014) 9:683–8. doi: 10.2147/cia.S57294, PMID: 24790420 PMC3998847

[27] Lindahl SH. Reviewing the options for local estrogen treatment of vaginal atrophy. Int J Womens Health. (2014) 6:307–12. doi: 10.2147/ijwh.S52555, PMID: 24648775 PMC3958523

[28] Van Gerwen OT Smith SE Muzny CA. Bacterial vaginosis in postmenopausal women. Curr Infect Dis Rep. (2023) 25:7–15. doi: 10.1007/s11908-022-00794-1, PMID: 37601955 PMC10438897

[29] Stoler MH Vichnin MD Ferenczy A Ferris DG Perez G Paavonen J. The accuracy of colposcopic biopsy: analyses from the placebo arm of the Gardasil clinical trials. Int J Cancer. (2011) 128:1354–62. doi: 10.1002/ijc.25470, PMID: 20506504

[30] Nam K Kim JKJ Jeon S. Human papillomavirus type 16 causes larger colposcopic lesions than other HPV types in patients with grade 3 cervical intraepithelial neoplasia. J Low Genit Tract Dis. (2013) 17:1–5. doi: 10.1097/LGT.0b013e31825afd5b, PMID: 23222045

[31] Wright TC Jr Stoler MH Sharma A Zhang GL Behrens C Wright TL. Evaluation of HPV-16 and HPV-18 genotyping for the triage of women with high-risk HPV+ cytology-negative results. Am J Clin Pathol. (2011) 136:578–86. doi: 10.1309/ajcptus5exas6dkz, PMID: 21917680

[32] Baumann A Henriques J Selmani Z Meurisse A Lepiller Q Vernerey D. HPV16 load is a potential biomarker to predict risk of high-grade cervical lesions in high-risk HPV-infected women: a large longitudinal French hospital-based cohort study. Cancers. (2021) 13:4149. doi: 10.3390/cancers13164149 34439304 PMC8394477

[33] Olmedo-Nieva L Muñoz-Bello JO Martínez-Ramírez I Martínez-Gutiérrez AD Ortiz-Pedraza Y González-Espinosa C. RIPOR2 expression decreased by HPV-16 E6 and E7 oncoproteins: an opportunity in the search for prognostic biomarkers in cervical cancer. Cells. (2022) 11. doi: 10.3390/cells11233942, PMID: 36497200 PMC9740487

[34] Yeo-Teh NSL Ito Y Jha S. High-risk human papillomaviral oncogenes E6 and E7 target key cellular pathways to achieve oncogenesis. Int J Mol Sci. (2018) 19:1706. doi: 10.3390/ijms19061706, PMID: 29890655 PMC6032416

[35] Zappacosta R Ianieri MM Tinelli A Gustapane S D’Angelo C Gatta DM. Detection of residual/recurrent cervical disease after successful LEEP conization: the possible role of mRNA-HPV test. Curr Pharm Des. (2013) 19:1450–7. doi: 10.2174/1381612811319080012 PMID: 23016778

[36] Stuebs FA Dietl AK Behrens A Adler W Geppert C Hartmann A. Concordance rate of colposcopy in detecting cervical intraepithelial lesions. Diagnostics. (2022) 12:2436. doi: 10.3390/diagnostics12102436 36292125 PMC9600163

[37] Dan XXJ. Accuracy of colposcopic biopsy diagnosis of CIN and related factors of missed diagnosis of cervical cancer. Chin J Fam Plan. (2014) 8:544–6.

